# Identification of potential biomarkers associated with immune infiltration in the esophageal carcinoma tumor microenvironment

**DOI:** 10.1042/BSR20202439

**Published:** 2021-02-17

**Authors:** Zhicheng Wang, Meilin Chen, Yanbing Qiu, Yuqin Yang, Yumei Huang, Xiaoxu Li, Wenling Zhang

**Affiliations:** 1 Department of Laboratory Medicine, The Third Xiangya Hospital, Central South University, Changsha, Hunan, People’s Republic of China; 2 Department of Laboratory Medicine, Xiangya School of Medicine, Central South University, Changsha, Hunan, People’s Republic of China

**Keywords:** biomarker, COL1A2, differentially expressed genes, esophageal carcinoma, immune infiltration

## Abstract

Tumor immune cell infiltration was significantly correlated with the progression and the effect of immunotherapy in cancers including esophageal carcinoma (ESCA). However, no biomarkers were identified which were associated with immune infiltration in ESCA. In the present study, a total of 128 common differentially expressed genes (DEGs) were identified between esophageal squamous cell carcinomas (ESCC) and esophageal adenocarcinomas (EAC). The results of gene ontology (GO) enrichment and Reactome pathway analysis displayed that the up-regulated DEGs were mainly involved in the regulation of extracellular matrix (ECM), while the down-regulated DEGs were mainly involved in the regulation of cornification and keratinocyte differentiation. The most significant module of up-regulated DEGs was selected by Molecular Complex Detection (MCODE). Top ten similar genes of COL1A2 were explored, then validation and the prognostic analysis of these genes displayed that COL1A2, COL1A1, COL3A1, ZNF469 and Periostin (POSTN) had the prognostic value which were up-regulated in ESCA. The expressions of COL1A2 and its four similar genes were mainly correlated with infiltrating levels of macrophages and dendritic cells (DCs) and showed strong correlations with diverse immune marker sets in ESCA. To summarize, COL1A2 and its four similar genes were identified as the potential biomarkers associated with immune infiltration in ESCA. These genes might be applied to immunotherapy for ESCA.

## Introduction

As a kind of gastrointestinal cancer, esophageal carcinoma (ESCA) ranks seventh according to incidence (572000 new cases) and sixth in mortality totally (509000 deaths) in 2018 [1]. The progression and chemoresistance of ESCA are important factors that impact patient survival. For those patients with locally advanced ESCA, the 5-year survival rate is approximately 20%. The standard treatment includes chemotherapy or chemoradiotherapy with surgery [2]. In recent years, tumor immunologic therapies have also shown promising early results in ESCA. And tumor immune cell infiltration significantly correlates with the progression and the effect of immunotherapy in cancers [3,4]. However, the specific molecular mechanism about immunotherapy for ESCA yet remains unclear and some genes associated with immune infiltration are required to be found.

With the development of gene chip technology and sequencing technology, bioinformatics is becoming more and more important [5–7]. Gene Expression Omnibus (GEO) and The Cancer Genome Atlas (TCGA) are two open databases, which provide gene chip data and sequencing data about the cancers. A lot of studies were published for analyzing the data from GEO and TCGA in ESCA, including the profiles of long noncoding RNA (lncRNA), microRNA (miRNA), and messenger RNA (mRNA). Interleukin 8 (IL8) and C–X–C chemokine receptor type 7 (CXCR-7) were predicted to differentially express in ESCA tissues compared with normal esophageal tissues and significantly correlate to the prognosis of patients with ESCA [8]. Liu et al. constructed the network of lncRNA, miRNA, and mRNA and found eight lncRNAs including WDFY3-AS2, CASC8, UGDH-AS1, RAP2C-AS1, AC007128.1, AC016205.1, AC092803.2, and AC079949.2 correlated with overall survival of the patients with ESCA [9]. Zeng et al. built the regulatory networks of miRNA-TF and miRNA-gene and identified the top five miRNAs (miRNA-93, miRNA-21, miRNA-4746, miRNA-196a-1, and miRNA-196a-2) with diagnostic value [10]. However, the immunotherapy-related genes were certainly not identified in these studies in ESCA.

In the present study, the common differentically expressed genes (DEGs) were screened between esophageal squamous cell carcinomas (ESCC) and esophageal adenocarcinoma (EAC) by analyzing the datasets from GEO. Then the gene ontology (GO) analysis, Reactome pathway analysis, and protein–protein interaction (PPI) analysis of common DEGs were performed. Subsequently, COL1A2 with its similar genes were selected in Gene Expression Profiling Interactive Analysis (GEPIA) and validated in UALCAN, meanwhile the prognostic significance analysis was performed. Finally, the immune infiltration analysis was carried out to explain the immunological significance of COL1A2 with its similar genes. The findings in this report revealed the important role of COL1A2 with its similar genes in regulating immune infiltration and suggested that these genes might be munotherapy-related genes in ESCA.

## Materials and methods

### Selection of microarray data from GEO

Two microarray datasets (GSE20347 and GSE26886) were downloaded from GEO. GSE20347 was based on the GPL571 platform (Affymetrix Human Genome U133A 2.0 Array) which contained 34 samples, including 17 pairs of tumors and their matching normal adjacent tissues from ESCC patients. GSE26886 was based on the GPL570 platform (Affymetrix Human Genome U133 Plus 2.0 Array) which contained 40 samples, including 21 EAC samples and 19 normal esophageal squamous epithelium samples.

### Screening of common DEGs between ESCC and EAC

We downloaded the raw CEL data and standardized them by the Affy package of R language [11]. Then the differential gene analysis was carried out using the limma R package and genes with an adjusted *P*-value <0.01 and |log2 fold change (FC)| > 2 were considered DEGs [12]. The heatmaps and volcano plots of DEGs were displayed. The common DEGs of two datasets were integrated using an online web tool, Venny 2.1, and the common DEGs including up-regulated and down-regulated genes were saved for subsequent analysis.

### GO and Reactome pathway analysis of common DEGs

The GO PANTHER version 14.0 was used to perform GO functional and Reactome pathway analysis of the common DEGs [13]. And the GO functional analysis of integrated DEGs refers to three parts: biological process (BP), cell component (CC), and molecular function (MF). FDR < 0.05 was considered to have a statistically significant difference.

### PPI network and module analysis

We constructed a PPI network of the common DEGs using the Search Tool for the Retrieval of Interacting Genes (STRING), an online web tool for predicting the correlation of protein function [14]. And the PPI network was visualized by Cytoscape software [15]. Then, the most significant module of up-regulated DEGs was selected by Molecular Complex Detection (MCODE), which was a plug-in application of Cytoscape [16].

### Validation of COL1A2 and its similar genes in UALCAN

UALCAN can analyze cancer OMICS data which is a comprehensive, user-friendly, and interactive web tool [17]. The expressions of COL1A2 and its similar genes were validated in primary ESCA samples compared with normal esophageal squamous epithelium samples. *P*<0.05 was considered to indicate a statistically significant difference.

### Similar genes and prognostic significance analysis in GEPIA

GEPIA is a web-based tool used to deliver fast and customizable functionalities based on TCGA and GTEx data. Its primary functions included differential expression analysis, patient survival analysis, similar gene detection, and so on [18]. Similar genes and prognostic significance analysis were executed here to study our target genes and *P*<0.05 was considered to have a statistically significant difference.

### Immune infiltration analysis in Tumor Immune Estimation Resource

Tumor Immune Estimation Resource (TIMER) is an interactive web tool to comprehensively investigate the molecular characterization of tumor-immune interactions. Six major functional modules allow users to interactively explore the correlations between immune infiltrates and a wide range of factors, including gene expression, somatic mutations, and somatic copy number alterations, and so on [19]. The correlations between the expression levels of COL1A2 with its similar genes and the infiltration levels of various immune cells were analyzed in ESCA, including B cells, CD8^+^ T cells, CD4^+^ T cells, neutrophils, macrophages, and dendritic cells (DCs). And we also analyzed the relationships between the expression levels of COL1A2 with its similar genes and the immune marker sets including CD8^+^ T cell, T cell (general), B cell, monocytes, tumor-associated macrophages (TAMs), M1 macrophages, M2 macrophages, neutrophils, natural killer (NK) cells, DCs, T-helper (Th) cells, T-helper 17 (Th17) cells, follicular helper T (Tfh) cells, regulatory T cells (Tregs) and exhausted T cells based on previous studies [20–22].

## Results

### Identification of common DEGs between ESCC and EAC

**Figure 1:**
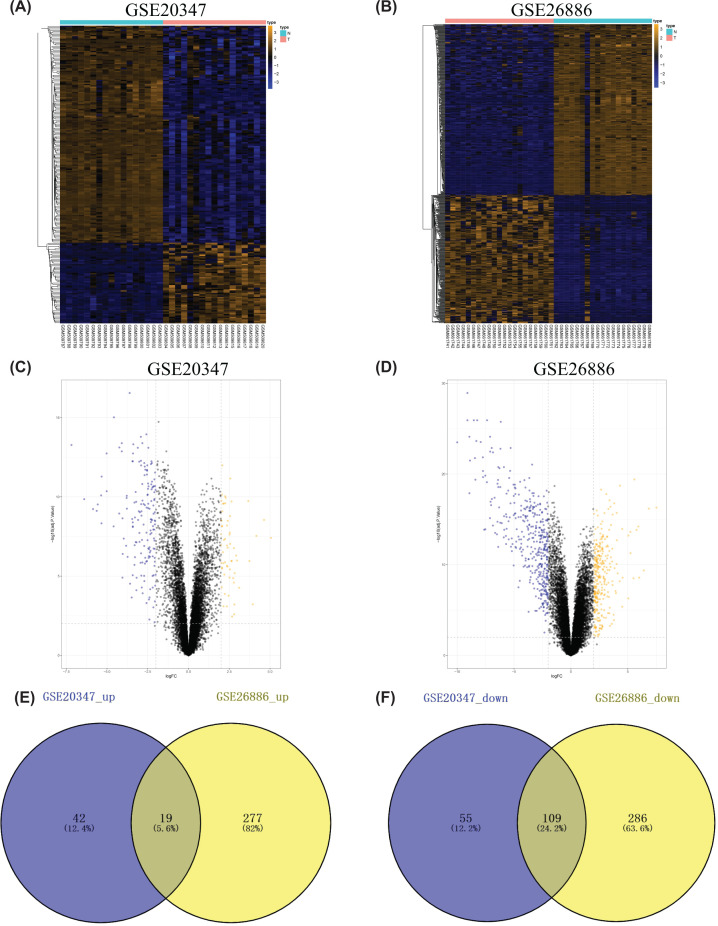
Identification of DEGs in GSE20347 and GSE26886 Heatmaps of DEGs in (**A**) GSE20347 and (**B**) GSE26886 were displayed. Volcano plots of DEGs in (**C**) GSE20347 and (**D**) GSE26886 were displayed. Orange: up-regulated DEGs; Blue: down-regulated DEGs; N: normal; T: tumor. (**E**) Up-regulated DEGs and (**F**) down-regulated DEGs were integrated between GSE20347 and GSE26886 by using the Venn diagram.

To find the common DEGs from the two subtypes of ESCA, we analyzed the ESCC chip expression dataset GSE20347 and EAC chip expression dataset GSE26886 (adjusted *P*<0.01 and |log2 FC| > 2). The GSE20347 dataset contained 225 DEGs, including 61 up-regulated genes and 164 down-regulated genes (Figure 1A,C). The GSE26886 dataset contained 691 DEGs, including 296 up-regulated genes and 395 down-regulated genes (Figure 1B,D). The common up-regulated DEGs and down-regulated DEGs from the two kinds of datasets were identified respectively, which were displayed by the Venn diagram, including 19 up-regulated genes and 109 down-regulated genes (Figure 1E,F).

### GO and reactome pathway analysis of common DEGs

**Figure 2:**
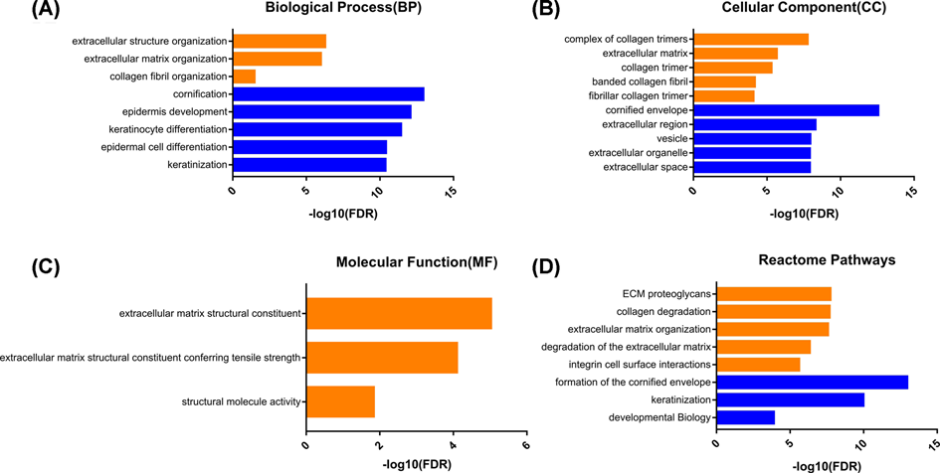
GO and Reactome pathway analysis of DEGs (**A**) BP, (**B**) CC, (**C**) MF, and (**D**) Reactome pathway enrichment analysis were displayed by the bar chart. The bars of up-regulated DEGs enrichment results were marked in orange and the bars of down-regulated DEGs enrichment results were marked in blue.

DEGs GO and Reactome pathway analysis were performed with PANTHER. The data shown in the figure needed to meet the following criteria: (1) the FDR < 0.05; (2) the top five significant enrichment results. We can find the BP of up-regulated genes which were mainly enriched in the extracellular structure organization, extracellular matrix (ECM) organization, and collagen fibril organization. The down-regulated genes were mainly enriched in the cornification, epidermis development, keratinocyte differentiation, epidermal cell differentiation, and keratinization (Figure 2A). And the CC of up-regulated genes were mainly enriched in the complex of collagen trimers, ECM, collagen trimer, banded collagen fibril, and fibrillar collagen trimer. The down-regulated genes were mainly enriched in the cornified envelope, extracellular region, vesicle, extracellular organelle, and extracellular space (Figure 2B). The MF of up-regulated genes were mainly enriched in the ECM structural constituent, ECM structural constituent conferring tensile strength and structural molecule activity, but the results of down-regulated genes were not displayed because the FDR > 0.05 (Figure 2C). As for the pathway analysis, the up-regulated DEGs were significantly enriched in the ECM proteoglycans, collagen degradation, ECM organization, degradation of the ECM, and integrin cell surface interactions, while the down-regulated DEGs were significantly enriched in the formation of the cornified envelope, keratinization, and developmental biology (Figure 2D).

### Construction of PPI network and module analysis

**Figure 3:**
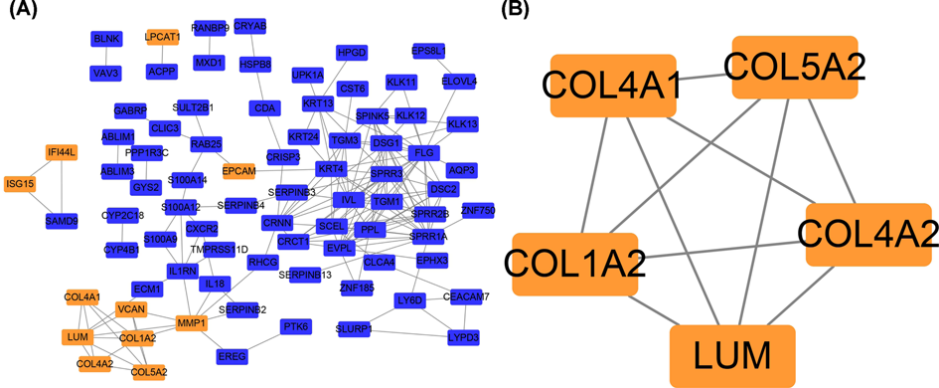
Construction of PPI network of DEGs and identification of the most significant module of up-regulated DEGs (**A**) PPI network of DEGs. The up-regulated genes were marked in orange and the down-regulated genes were marked in blue. (**B**) The most significant module of up-regulated DEGs. The up-regulated genes were marked in orange.

The PPI network of DEGs was constructed by using the Cytoscape, which contained 81 nodes and 174 edges (Figure 3A). The most significant module of up-regulated DEGs was identified by using MCODE, which contained COL1A2, COL5A2, COL4A2, LUM, and COL4A1 (Figure 3B).

### Prognostic significance analysis and validation of COL1A2 and its similar genes

**Figure 4:**
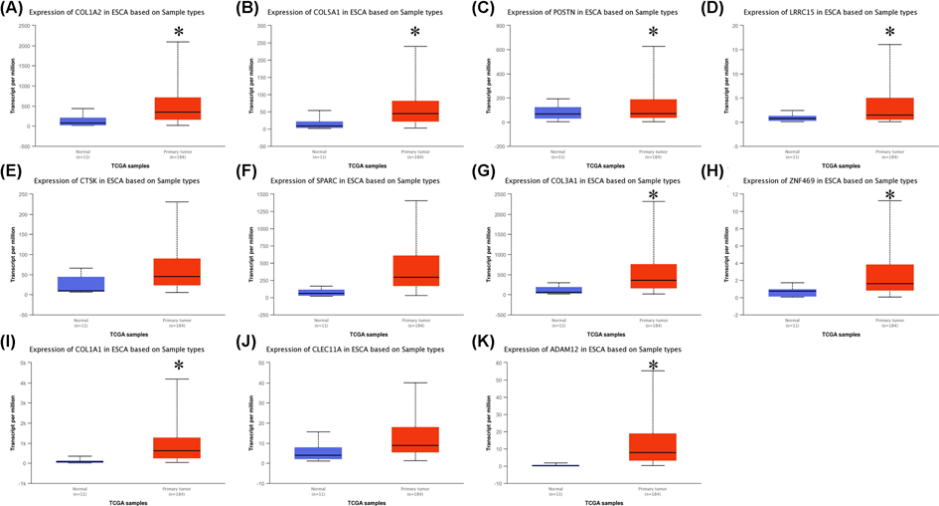
Boxplots of mRNA expression levels of COL1A2 and its similar genes in normal esophageal squamous epithelium samples and primary ESCA samples (**A**) COL1A2 (**B**) COL5A1 (**C**) POSTN (**D**) LRRC15 (**E**) CTSK (**F**) SPARC (**G**) COL3A1 (**H**) ZNF469 (**I**) COL1A1 (**J**) CLEC11A (**K**) ADAM12. Normal, normal esophageal squamous epithelium samples; Primary tumor, primary ESCA samples. *Normal vs Primary tumor and *P*<0.05. *P*<0.05 was considered statistically significant.

**Figure 5:**
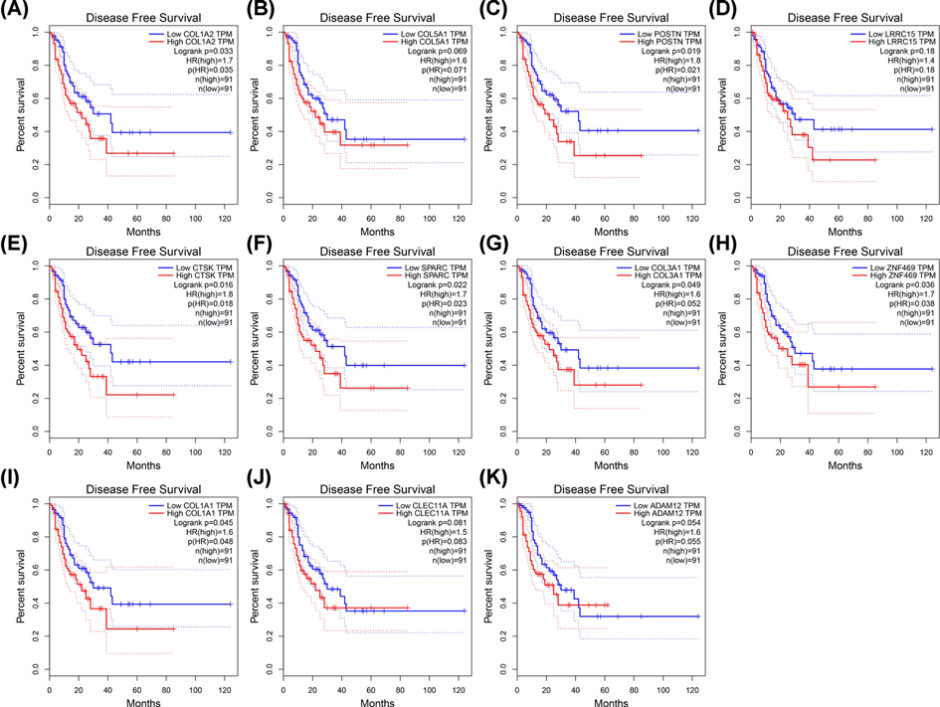
DFS curves of COL1A2 and its similar genes in ESCA from the TCGA database (**A**) COL1A2 (**B**) COL5A1 (**C**) POSTN (**D**) LRRC15 (**E**) CTSK (**F**) SPARC (**G**) COL3A1 (**H**) ZNF469 (**I**) COL1A1 (**J**) CLEC11A (**K**) ADAM12. *P*<0.05 was considered statistically significant.

COL1A2 was a subtype of Type I collagen, the pieces of evidence suggest its mRNA expression in ESCC tumors was higher than that in normal tissues. And it was associated with the survival of ESCC patients [23]. In the following study, we selected COL1A2 for our important target gene. In the first place, we explored the top ten similar genes of COL1A2 in ESCA by using GEPIA (Table 1). In order to validate our results, we analyzed the differential expression of COL1A2 and its similar genes between 11 normal esophageal squamous epithelium samples and 184 primary ESCA samples by using UALCAN (Figure 4A–K). Except for CTSK, SPARC, and CLEC11A, all of the genes expressed higher in primary ESCA samples compared with normal esophageal squamous epithelium samples. Meanwhile, we investigated whether these genes contributed to the prognosis of ESCA patients, GEPIA was used to analyze the disease-free survival (DFS) of COL1A2 and its similar genes in patients. As shown in Figure 5A–K, the higher expression of COL1A2 and its similar genes showed worse DFS in ESCA patients, except for COL5A1, LRRC15, ADAM12 and CLEC11A. These genes that have been validated and had prognostic value will be used for later study.

**Table 1:** The similar genes of COL1A2 explored by GEPIA and ranked by Pearson correlation coefficient (PCC) in ESCA tumors

Genes symbol	Gene ID	PCC
*COL1A1*	ENSG00000108821.13	0.98
*COL3A1*	ENSG00000168542.12	0.97
*SPARC*	ENSG00000113140.10	0.93
*ZNF469*	ENSG00000225614.2	0.92
*POSTN*	ENSG00000133110.14	0.92
*CTSK*	ENSG00000143387.12	0.92
*COL5A1*	ENSG00000130635.15	0.91
*LRRC15*	ENSG00000172061.8	0.91
*ADAM12*	ENSG00000148848.14	0.90

### The expression levels of COL1A2 and its similar genes correlate with the infiltration levels of immune cells in ESCA

**Figure 6:**
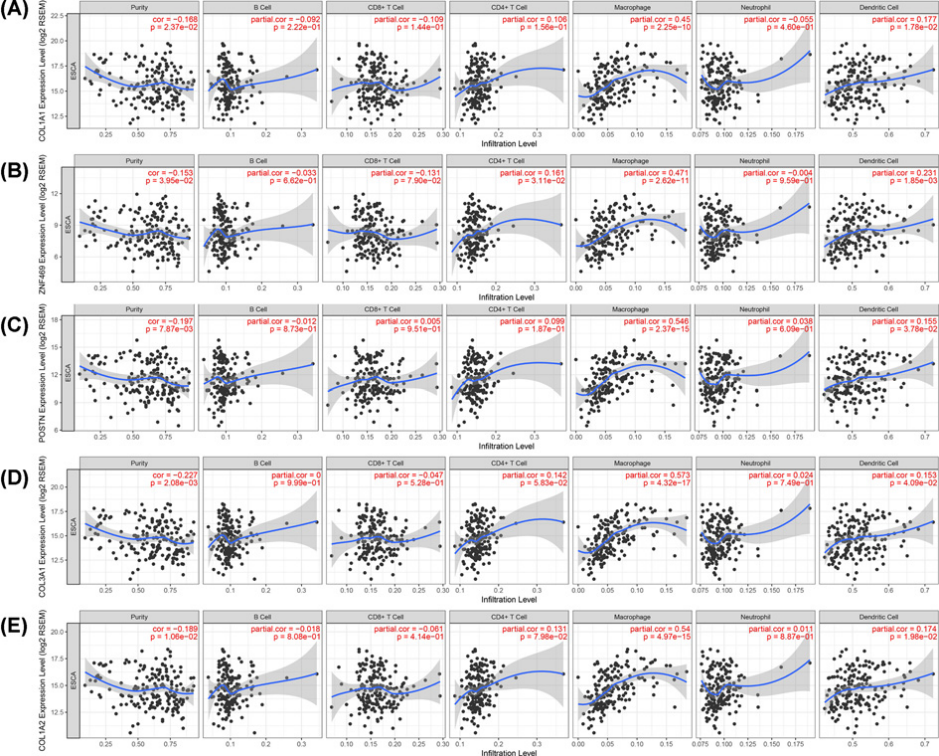
Correlation between the expression levels of COL1A2 with its similar genes and immune infiltration level in ESCA (**A**) COL1A1 (**B**) ZNF469 (**C**) POSTN (**D**) COL3A1 (**E**) COL1A2. *P*<0.05 was considered statistically significant.

The infiltration of immune cells such as TAMs and Tregs in the microenvironment can promote esophageal carcinogenesis [24]. Therefore, we explored the relationship between the expression of COL1A2 with its similar genes and the infiltrating immune cells in ESCA using the TIMER database. Tumor purity plays an important role in the analysis of immune cell infiltration about clinical tumor samples by genomic approaches [25]. After adjusting the analysis by tumor purity, we found that the expression levels of COL1A2, COL1A1, COL3A1, ZNF469 and Periostin (POSTN) were significantly correlated with tumor purity in ESCA. And the expression levels of these genes were positively correlated with the infiltration levels of macrophages and DCs, as well as the expression levels of ZNF469 was also positively correlated with the infiltration levels of CD4^+^ T cells (Figure 6).

### Correlation analysis between mRNA levels of COL1A2 with its similar genes and immune marker sets

To investigate the relationship between COL1A2 with its similar genes and the different immune infiltrating cells, the correlations between COL1A2 with its similar genes and immune marker sets of various immune cells of ESCA were explored in the TIMER database. We analyzed the correlations between the expression of COL1A2 with its similar genes and immune marker genes of diverse immune cells, including CD8^+^ T cells, T cells (general), B cells, monocytes, TAMs, M1 and M2 macrophages, neutrophils, NK cells, and DCs in ESCA. Besides, the diverse T cells, for instance, Th1 cells, Th2 cells, Tfh cells, Th17 cells, Tregs, and exhausted T cells were also analyzed. Before the correlation analysis, we adjusted it for tumor purity because the tumor purity of clinical samples can impact the immune infiltration analysis. As shown in Table 2, the expression of COL1A2 was significantly correlated with the expression of marker genes from tumor-infiltrating B cells (CD19 and CD79A), monocytes (CD86 and CD115), TAMs (CCL2 and IL10), M1 macrophages (COX2), neutrophils (CD66b, CD11b, and CCR7), DCs (BDCA-4), T-helper (STAT4, GATA3, STAT6, BCL6, and IL17A), Treg (FOXP3, CCR8, and TGFβ), and exhausted T cells (CTLA4 and TIM-3). The expression of COL1A1 was significantly correlated with the expression of marker genes from tumor-infiltrating CD8^+^ T cell (CD8B), B cells (CD19 and CD79A), monocytes (CD86 and CD115), TAMs (CCL2 and IL10), M1 macrophages (COX2), neutrophils (CD66b, CD11b, and CCR7), DCs (HLA-DPA1), T-helper (STAT4, STAT5A, STAT6, BCL6, and IL17A), Treg (FOXP3, CCR8, and TGFβ), and exhausted T cells (TIM-3). The expression of COL3A1 was significantly correlated with the expression of marker genes from tumor-infiltrating B cells (CD19 and CD79A), monocytes (CD86 and CD115), M1 macrophages (COX2), neutrophils (CD66b and CD11b), DCs (HLA-DQB1 and HLA-DPA1), T-helper (STAT4, GATA3, STAT6, BCL6, and IL17A), Treg (FOXP3, CCR8, and TGFβ), and exhausted T cells (CTLA4 and TIM-3). The expression of ZNF469 was significantly correlated with the expression of marker genes from tumor-infiltrating B cells (CD19 and CD79A), monocytes (CD86 and CD115), neutrophils (CD66b and CD11b), T-helper (STAT4, STAT6, STAT5A, BCL6, and IL17A), Treg (FOXP3, CCR8, and TGFβ), and exhausted T cells (CTLA4 and TIM-3). The expression of POSTN was significantly correlated with the expression of marker genes from tumor-infiltrating B cells (CD19 and CD79A), monocytes (CD86 and CD115), TAMs (CD68), M1 macrophages (COX2), neutrophils (CD66b, CD11b, and CCR7), T-helper (STAT4, GATA3, STAT6, STAT5A, BCL6, and IL17A), Treg (FOXP3, CCR8, and TGFβ) and exhausted T cells (CTLA4 and TIM-3). All of the results demonstrated that COL1A2 with its similar genes were correlated with the infiltration of the immune cells in ESCA.

**Table 2:** Correlation analysis between COL1A2 with its similar genes and related genes and markers of immune cells in TIMER

Description	Gene markers	COL1A2		COL1A1		COL3A1		ZNF469		POSTN	
		Purity		Purity		Purity		Purity		Purity	
		Cor	*P*	Cor	*P*	Cor	*P*	Cor	*P*	Cor	*P*
CD8^+^ T cell	CD8A	−0.070	0.353	−0.127	0.090	−0.055	0.466	−0.031	0.681	−0.044	0.561
	CD8B	−0.133	0.076	−0.197	†	−0.112	0.134	−0.102	0.171	−0.113	0.132
T cell (general)	CD3D	−0.063	0.399	−0.116	0.122	−0.045	0.544	−0.050	0.507	−0.068	0.363
	CD3E	−0.042	0.573	−0.094	0.207	−0.025	0.741	−0.036	0.628	−0.035	0.640
	CD2	−0.048	0.525	−0.101	0.178	−0.031	0.675	−0.030	0.691	−0.025	0.744
B cell	CD19	−0.156	*	−0.231	†	−0.157	*	−0.155	*	−0.158	*
	CD79A	−0.181	*	−0.259	‡	−0.153	*	−0.193	†	−0.156	*
Monocyte	CD86	0.447	‡	0.405	‡	0.455	‡	0.479	‡	0.446	‡
	CD115	0.408	‡	0.333	‡	0.431	‡	0.395	‡	0.394	‡
TAM	CCL2	0.482	‡	0.419	‡	0.202	0.086	0.112	0.346	0.212	0.072
	CD68−	−0.003	0.972	−0.018	0.812	0.203	0.085	−0.134	0.258	0.490	‡
	IL10	0.387	‡	0.351	‡	0.215	0.067	0.217	0.065	0.083	0.485
M1 Macrophage	INOS	0.223	0.058	0.128	0.280	0.211	0.073	0.219	0.063	0.226	0.054
	IRF5	−0.027	0.822	−0.093	0.433	−0.147	0.214	−0.014	0.908	0.015	0.901
	COX2	0.382	‡	0.369	†	0.413	‡	0.147	0.216	0.355	†
M2 Macrophage	CD163	0.140	0.237	0.039	0.740	0.095	0.426	0.086	0.471	0.061	0.610
	VSIG4	0.163	0.168	0.093	0.434	0.090	0.449	0.064	0.591	0.055	0.645
	MS4A4A	0.111	0.348	−0.005	0.967	0.055	0.642	−0.027	0.819	0.130	0.273
Neutrophils	CD66b	−0.195	†	−0.209	†	−0.185	*	−0.202	†	−0.200	†
	CD11b	0.324	‡	0.244	‡	0.335	‡	0.302	‡	0.305	‡
	CCR7	−0.154	*	−0.241	†	−0.134	0.072	−0.124	0.098	−0.161	*
NK cell	KIR2DL1	0.016	0.828	0.021	0.777	0.019	0.805	−0.006	0.939	−0.035	0.640
	KIR2DL3	−0.036	0.629	−0.031	0.682	−0.022	0.768	−0.003	0.969	0.001	0.990
	KIR2DL4	−0.115	0.125	−0.121	0.106	−0.105	0.160	−0.079	0.293	−0.105	0.162
	KIR3DL1	−0.010	0.898	−0.023	0.754	−0.022	0.769	0.048	0.524	−0.067	0.372
	KIR3DL2	0.006	0.938	0.017	0.817	0.002	0.978	0.005	0.949	−0.005	0.946
	KIR3DL3	−0.034	0.654	−0.034	0.651	−0.023	0.762	−0.051	0.494	−0.048	0.526
	KIR2DS4	−0.003	0.971	−0.012	0.869	−0.013	0.868	0.022	0.765	−0.049	0.516
DC	HLA-DPB1	−0.045	0.708	−0.188	0.111	−0.165	0.163	−0.081	0.495	−0.041	0.731
	HLA-DQB1	−0.152	0.199	−0.222	0.059	−0.254	*	−0.108	0.365	0.057	0.635
	HLA-DRA	−0.028	0.815	−0.138	0.244	−0.104	0.380	−0.093	0.436	−0.057	0.633
	HLA-DPA1	−0.115	0.332	−0.246	*	−0.234	*	−0.161	0.173	0.012	0.918
	BDCA-1	0.058	0.625	−0.155	0.191	0.031	0.792	−0.084	0.480	0.209	0.077
	BDCA-4	0.245	*	0.185	0.117	0.186	0.116	0.134	0.260	0.058	0.626
	CD11c	0.027	0.820	−0.055	0.645	−0.048	0.689	0.023	0.848	−0.047	0.692
Th1	T-bet	−0.028	0.708	−0.083	0.269	−0.013	0.862	0.023	0.755	−0.005	0.952
	STAT4	0.238	†	0.172	*	0.276	‡	0.238	†	0.292	‡
	STAT1	−0.005	0.947	−0.020	0.794	0.016	0.834	0.041	0.587	0.048	0.522
	IFN-γ	0.017	0.817	−0.001	0.990	0.024	0.745	0.061	0.415	0.008	0.918
	TNF-α	0.041	0.581	0.063	0.400	0.036	0.636	0.112	0.133	0.018	0.811
Th2	GATA3	0.154	*	0.140	0.061	0.158	*	0.126	0.092	0.154	*
	STAT6	−0.311	‡	−0.298	‡	−0.298	‡	−0.270	‡	−0.282	‡
	STAT5A	−0.139	0.063	−0.171	*	−0.114	0.127	−0.160	*	−0.154	*
	IL13	0.091	0.227	0.047	0.530	0.109	0.143	0.076	0.309	0.055	0.462
Tfh	BCL6	0.288	‡	0.280	‡	0.282	‡	0.344	‡	0.294	‡
	IL21	0.019	0.802	0.002	0.977	0.022	0.768	0.064	0.394	0.066	0.381
Th17	STAT3	−0.126	0.091	−0.143	0.055	−0.121	0.105	−0.129	0.085	−0.103	0.171
	IL17A	−0.253	‡	−0.249	‡	−0.225	†	−0.227	†	−0.275	‡
Treg	FOXP3	0.287	‡	0.200	†	0.296	‡	0.297	‡	0.286	‡
	CCR8	0.302	‡	0.213	†	0.323	‡	0.303	‡	0.309	‡
	STAT5B	0.107	0.152	0.032	0.669	0.123	0.101	0.123	0.101	0.105	0.160
	TGFβ	0.395	‡	0.405	‡	0.363	‡	0.449	‡	0.367	‡
T cell exhaustion	PD-1	0.016	0.829	−0.032	0.665	0.030	0.687	0.062	0.409	0.018	0.812
	CTLA4	0.184	*	0.119	0.112	0.195	†	0.202	†	0.189	*
	LAG3	0.091	0.225	0.053	0.482	0.098	0.192	0.143	0.055	0.091	0.225
	TIM-3	0.426	‡	0.370	‡	0.441	‡	0.445	‡	0.425	‡
	GZMB	0.005	0.952	−0.009	0.900	−0.002	0.976	0.070	0.351	-0.014	0.850

Abbreviations: Cor, R value of Spearman’s correlation; Purity, correlation adjusted by purity.

*P<0.05.

^†^*P<0.01.*

^‡^*P<0.001.*

## Discussion

Over the past decade, there are many studies about bioinformatics analysis that have been published to explore the potential pathological mechanisms in ESCA. However, the mechanism of immune cell infiltration and the genes associated with immune infiltration in ESCA yet remain unclear. We firstly analyzed two subtypes of ESCA respectively, which contained the datasets of ESCC and EAC from GEO to find the common DEGs from the different histologic subtypes. Altogether 128 DEGs including 19 up-regulated genes and 109 down-regulated genes were screened. To demonstrate the functional mechanism of common DEGs in ESCA, we performed GO enrichment and Reactome pathway analysis. The results of GO enrichment and Reactome pathway analysis displayed that the up-regulated DEGs were mainly involved in the regulation of ECM such as extracellular structure organization and ECM organization, while the down-regulated DEGs were mainly involved in the regulation of cornification and keratinocyte differentiation. These results suggest the major variations in ESCA were involved in the remodeling of the tumor microenvironment (TME) and cell differentiation. The TME is an internal and complex environment in the tumor, which includes all kinds of ECM (for instance, collagen and laminin) and stromal cells including fibroblasts, immune cells, and vascular endothelial cells [26,27]. During tumor progression, the TME interacts with tumor cells depending on different kinds of immune cells (for example, DCs, macrophages, and myeloid-derived suppressor cells) to mediate their immunologic tolerance, subsequently impacting the clinical effect of immunotherapy [28,29].

Aiming to find the most significant module involved in immune infiltration, we constructed the PPI network of DEGs and identified the most significant module of up-regulated DEGs by using MCODE. The most significant module of up-regulated DEGs contained COL1A2, COL5A2, COL4A2, LUM, and COL4A1. One of five up-regulated genes, COL1A2, is a subtype of Type I collagen, which can be produced by stromal fibroblasts and cancer cells [30–32]. And COL1A2 plays an important role in the invasion and metastasis of ovarian cancer cells since it promotes the migration of cancer cells by interacting with α2β1-integrin [33]. However, what roles it plays in immune cell infiltration and immunotherapy about ESCA are still indistinct.

Subsequently, we explored the similar genes of COL1A2 in ESCA by using GEPIA. The top ten similar genes were screened including COL1A1, COL3A1, SPARC, ZNF469, POSTN, CTSK, COL5A1, LRRC15, ADAM12, and CLEC11A. We validated the expressions of COL1A2 and its similar genes in primary ESCA samples compared with normal esophageal squamous epithelium samples, while CTSK, SPARC, and CLEC11A were not significantly up-regulated. Among these genes, the high expression of COL1A2, COL1A1, COL3A1, SPARC, ZNF469, POSTN, and CTSK were negatively correlated with the DFS of ESCA patients. Then these genes (COL1A2, COL1A1, COL3A1, ZNF469, and POSTN) that have been validated and had prognostic value were used for immune-related research. The expression levels of COL1A2, COL1A1, COL3A1, ZNF469, and POSTN were positively correlated with the infiltration levels of macrophages and DCs, and the expression levels of ZNF469 was also positively correlated with the infiltration levels of CD4^+^ T cells. These results indicated these genes might be the candidate genes for assessing the immune infiltration levels in ESCA.

Finally, to further explore the relationship between COL1A2 with its similar genes and the infiltration levels of various immune cells comprehensively, we analyzed the correlation between these genes and immune marker sets. The expression of COL1A2 was significantly correlated with the expression of marker genes from tumor-infiltrating B cells (CD19 and CD79A), monocytes (CD86 and CD115), TAMs (CCL2 and IL10), M1 macrophages (COX2), neutrophils (CD66b, CD11b, and CCR7), DCs (BDCA-4), T-helper (STAT4, GATA3, STAT6, BCL6, and IL17A), Treg (FOXP3, CCR8, and TGFβ), and exhausted T cells (CTLA4 and TIM-3). The expression of COL1A1 was significantly correlated with the expression of marker genes from tumor-infiltrating CD8^+^ T cell (CD8B), B cells (CD19 and CD79A), monocytes (CD86 and CD115), TAMs (CCL2 and IL10), M1 macrophages (COX2), neutrophils (CD66b, CD11b, and CCR7), DCs (HLA-DPA1), T-helper (STAT4, STAT5A, STAT6, BCL6, and IL17A), Treg (FOXP3, CCR8, and TGFβ), and exhausted T cells (TIM-3). Type I collagen (COL1A1 and COL1A2) was correlated with the resembling of TAMs in non-small cell lung carcinoma (NSCLC) while it was not correlated with the paucity of T-cell accumulation in pancreatic ductal adenocarcinoma (PDAC), which were consistent with our results [34,35]. The expression of COL3A1 was significantly correlated with the expression of marker genes from tumor-infiltrating B cells (CD19 and CD79A), monocytes (CD86 and CD115), M1 macrophages (COX2), neutrophils (CD66b and CD11b), DCs(HLA-DQB1 and HLA-DPA1), T-helper (STAT4, GATA3, STAT6, BCL6, and IL17A), Treg (FOXP3, CCR8, and TGFβ), and exhausted T cells (CTLA4 and TIM-3). COL3A1, a subtype of Type III collagen, which is similar to Type I collagen, and primarily refers to the growth and metastasis of tumors such as osteosarcoma and nasopharyngeal tumors [36,37]. While our results suggested that COL3A1 also can be a potential biomarker for assessing the infiltration of the immune cells in ESCA. The high expression of COL3A1 may promote the growth and metastasis of ESCA by recruiting various immune cells. The expression of ZNF469 was significantly correlated with the expression of marker genes from tumor-infiltrating B cells (CD19 and CD79A), monocytes (CD86 and CD115), neutrophils (CD66b and CD11b), T-helper (STAT4, STAT6, STAT5A, BCL6, and IL17A), Treg (FOXP3, CCR8, and TGFβ), and exhausted T cells (CTLA4 and TIM-3). Zinc finger protein (ZNF469) gene mainly refers to the pathological process of cornea-related disease and it is similar to three types of collagen including COL1A1, COL1A2, and COL4A1 [38–40]. Interestingly, we firstly found the important roles of ZNF469 in the assessment of prognosis and immune cell infiltration in ESCA. This gene may be an important biomarker associated with immune infiltration in the ESCA TME. The expression of POSTN was significantly correlated with the expression of marker genes from tumor-infiltrating B cells (CD19 and CD79A), monocytes (CD86 and CD115), TAMs (CD68), M1 macrophages (COX2), neutrophils (CD66b, CD11b, and CCR7), T-helper (STAT4, GATA3, STAT6, STAT5A, BCL6, and IL17A), Treg (FOXP3, CCR8, and TGFβ), and exhausted T cells (CTLA4 and TIM-3). POSTN is a soluble ECM protein and it is up-regulated in many cancers such as ESCA, head and neck squamous cell carcinoma, and non-small cell lung cancer [41–43]. Recent studies demonstrated that it was an important biomarker associated with the pathogenesis and prognosis of ESCA, and our results further underlined the important role of POSTN in immune cells infiltration [44,45].

Different from previous studies, we analyzed the common DEGs between the ESCC and EAC to find the most important variations in ESCA and identified the important roles of COL1A2 with its similar genes in influencing the immune infiltration in ESCA. However, some limitations still exist in the present study. Firstly, some technical defects of gene chip and sequencing technology existed here. Secondly, we only analyzed the potential biomarker in ESCA and the specific mechanism of immune cell infiltration induced by these genes are needed to be demonstrated in vivo and in vitro experiments.

In summary, we identified COL1A2 and its similar genes as the potential biomarkers for assessing the infiltration levels of immune cells using a comprehensive analysis of bioinformatics, which could help us better understand the mechanism of tumor immune cell infiltration in ESCA. However, more studies are needed to verify these results.

## Data Availability

The gene expression data are available in public databases including GEO (https://www.ncbi.nlm.nih.gov/geo/) and TCGA (https://portal.gdc.cancer.gov).

## Abbreviations

BP: biological process
CC: cell component
COL1A2: collagen type I alpha 2 chain
DC: dendritic cell
DEG: differentially expressed gene
DFS: disease-free survival
EAC: esophageal adenocarcinoma
ECM: extracellular matrix
ESCA: esophageal carcinoma
ESCC: esophageal squamous cell carcinoma
FC: fold change
FDR: false discovery rate
GEO: Gene Expression Omnibus
GEPIA: Gene Expression Profiling Interactive Analysis
GO: gene ontology
GTEx: Genotype-Tissue Expression
lncRNA: long noncoding RNA
MF: molecular function
miRNA: microRNA
mRNA: messenger RNA
NK: natural killer
POSTN: Periostin
PPI: protein–protein interaction
TAM: tumor-associated macrophage
TCGA: The Cancer Genome Atlas
Tfh: follicular helper T
Th: T-helper
Th17: T-helper 17
TIMER: Tumor Immune Estimation Resource
TME: tumor microenvironment
Treg: regulatory T cell
